# Chlorogenic Acid Potentiates the Anti-Inflammatory Activity of Curcumin in LPS-Stimulated THP-1 Cells

**DOI:** 10.3390/nu12092706

**Published:** 2020-09-04

**Authors:** Akshay Bisht, Martin Dickens, Kay Rutherfurd-Markwick, Rohith Thota, Anthony N. Mutukumira, Harjinder Singh

**Affiliations:** 1 School of Food and Advanced Technology, College of Science, Massey University, Auckland 0745, New Zealand; bishtakshay51@gmail.com (A.B.); A.N.Mutukumira@massey.ac.nz (A.N.M.); 2 School of Health Sciences, College of Health, Massey University, Auckland 0745, New Zealand; K.J.Rutherfurd@massey.ac.nz; 3 Riddet Research Institute, Massey University, Palmerston North 4442, New Zealand; R.Thota@massey.ac.nz (R.T.); H.Singh@massey.ac.nz (H.S.); 4 School of Biomedical Sciences and Pharmacy, University of Newcastle, Callaghan, NSW 2308, Australia

**Keywords:** curcumin, chlorogenic acid, bioactive combination, inflammation, NF-κB pathway

## Abstract

The anti-inflammatory effects of curcumin are well documented. However, the bioavailability of curcumin is a major barrier to its biological efficacy. Low-dose combination of complimentary bioactives appears to be an attractive strategy for limiting barriers to efficacy of bioactive compounds. In this study, the anti-inflammatory potential of curcumin in combination with chlorogenic acid (CGA), was investigated using human THP-1 macrophages stimulated with lipopolysaccharide (LPS). Curcumin alone suppressed TNF-α production in a dose-dependent manner with a decrease in cell viability at higher doses. Although treatment with CGA alone had no effect on TNF-α production, it however enhanced cell viability and co-administration with curcumin at a 1:1 ratio caused a synergistic reduction in TNF-α production with no impact on cell viability. Furthermore, an qRT-PCR analysis of NF-κB pathway components and inflammatory biomarkers indicated that CGA alone was not effective in reducing the mRNA expression of any of the tested inflammatory marker genes, except TLR-4. However, co-administration of CGA with curcumin, potentiated the anti-inflammatory effects of curcumin. Curcumin and CGA together reduced the mRNA expression of pro-inflammatory cytokines [TNF-α (~88%) and IL-6 (~99%)], and COX-2 (~92%), possibly by suppression of NF-κB (~78%), IκB-β-kinase (~60%) and TLR-4 receptor (~72%) at the mRNA level. Overall, co-administration with CGA improved the inflammation-lowering effects of curcumin in THP-1 cells.

## 1. Introduction

Inflammation is a vital protective response of the body characterised by the synthesis and release of pro-inflammatory cytokines, such as tumour necrosis factor-α (TNF-α), and interleukin-6 (IL-6), upregulation of enzymes (e.g., cyclooxygenase-2 (COX-2) and inducible nitric oxide synthase (iNOS)), and stimulation of intracellular signalling pathways, particularly those activating nuclear factor kappa-B (NF-κB), a transcription factor that orchestrates inflammatory responses in many tissues [1,2,3]. A long-term, uncontrolled, low-grade inflammatory response is involved in many chronic conditions, such as obesity, diabetes, bronchitis, pancreatitis, colitis, and cardiovascular disease, as well as some types of cancer [2,4,5,6].

Several anti-inflammatory drugs have been developed to control chronic inflammation by reducing the production of inflammatory mediators, but long-term consumption of these drugs may have side effects, such as bleeding in the stomach and predisposition to ulcers [7]. Therefore, there is a growing interest in alternative treatments that are natural and can be a part of the diet [8]. Traditionally, plant-based diets and their derivatives have been used to prevent inflammation and related chronic diseases [9]. The therapeutic effect of food is primarily associated with the presence of naturally occurring bioactive compounds present in fruit, vegetables, and grains [10,11]. Some of these compounds can suppress inflammation and reduce oxidative stress in cells, either by modulation of the signalling pathways involved, direct scavenging of oxidants or induction of antioxidant enzymes, thus helping avert chronic inflammation-related diseases [12,13,14].

Curcumin, the principal phenolic compound extracted from the rhizome of the turmeric plant, is one such bioactive that has been extensively studied for its anti-inflammatory health benefits. Curcumin is reported to affect multiple inflammation-associated biomarkers, including transcription factors, enzymes, pro-inflammatory cytokines and chemokines, and free radicals [15,16,17]. In vitro studies with curcumin treatment have shown to downregulate TNF-α, IL-6, IL-1β, nitric oxide (NO), prostaglandin E_2_ (PGE_2_), an inhibitor of κB subunit (IκB) and IκB kinase (IKK) via suppressing the NF-κB signalling pathway [18,19,20,21]. Administration of curcumin (5–30 µM) to rat vascular smooth muscle cells stimulated with LPS (1 µg/mL) to induce inflammation, resulted in decreases in expression of TNF-α, NO, iNOS and IκBα by suppressing the mitogen-activated protein kinase (MAPK) and NF-κB pathways [22]. Similar anti-inflammatory effects of curcumin are reported in animal and clinical trials. Curcumin (100 mg/kg bodyweight for 30 days) decreased the secretion and transcription of TNF-α, IL-6, IL-1β and monocyte chemoattractant protein-1 (MCP-1) in adult male Wistar rats [23]. Supplementation of 100 and 200 mg/kg bodyweight curcumin for three days downregulated TNF-α, IL-6, IL-1β, IL-8, IκBα by inhibiting MAPK/NF-κB pathways in female BALB/c and C57BL/6 mice [24]. Similar reductions in the above-mentioned anti-inflammatory biomarkers are reported in several clinical trials at a dose ranging 500–1000 mg per day [6,25,26]. However, the low bioavailability of curcumin has proved a challenge, requiring high doses to deliver its intended benefits in humans. Previous studies have reported that a minimum dosage of 3.6 g/day of curcumin is required to achieve measurable plasma levels in humans [27,28]. This problem has led to the search for other bioactive compounds that may act synergistically with curcumin to achieve anti-inflammatory effects at lower doses.

Although less studied than curcumin, chlorogenic acid (CGA), an ester of cinnamic acids with quinic acid obtained primarily from coffee and several types of fruit, is a potent antioxidant that can mitigate unbalanced intracellular redox state [29,30]. In vitro studies have also reported the anti-inflammatory effect of CGA predominantly by scavenging reactive oxygen species (ROS) and reactive nitrogen species (RNS) [31,32]. A decrease in intercellular oxidative stress will reduce the activation of the NF-κB signalling pathway, thereby showing an anti-inflammatory response. It is hypothesised that co-administration of an antioxidant CGA with curcumin, may improve the stability of curcumin and thereby enhance the anti-inflammatory benefits of curcumin at a lower dose. To test if administration of curcumin with CGA might result in enhanced anti-inflammatory properties, herein, we have investigated the effect of curcumin and CGA, both individually and in combination, on LPS-mediated inflammation in human leukaemia monocyte (THP-1) cells.

## 2. Materials and Methods

### 2.1. Chemicals and Reagents

Phorbol 12-myristate 13-acetate (PMA, Cat. # P1585), lipopolysaccharide (LPS, Cat. # L5293), 3-(4,5-Dimethylthiazol-2-Yl)-2,5-Diphenyltetrazolium Bromide (MTT, Cat. # M5655), curcumin (Cat. # 08511) and CGA (Cat. # 500590) were purchased from Sigma-Aldrich, Auckland, New Zealand. Roswell Park Memorial Institute RPMI-1640 (Cat. # 72400120) and penicillin/streptomycin solution (Cat. # 15070063) from ThermoFisher Scientific, Auckland, NZ. Fetal bovine serum (FBS, Cat. # MG-FBS0820) from MediRay, Auckland, New Zealand. Stock solutions of curcumin (20 mM) and CGA (50 mM) were prepared in DMSO, and 100-µL aliquots were stored at −80 °C until required for use.

### 2.2. Cell Culture and Cell Treatments

THP-1 cells were purchased from Sigma-Aldrich, NZ (Cat. # 88081201) and cultured in RPMI 1640 medium supplemented with 10% FBS and 1% penicillin/streptomycin at 37 °C in 5% CO_2_. THP-1 cells, at a density of 0.5 × 10^6^ cells / well in 6-well plates, were differentiated into macrophages by treatment with 50 nM PMA for 72 h. Differentiated THP-1 cells were then treated with curcumin and CGA for 1 h, both individually and in combination, at a variety of concentrations (1–25 µM) as indicated in the figure legends. The cells, in the presence of bioactive compounds, were then incubated with LPS (100 ng/mL) for another 4 h to induce an inflammatory response. Cells were pre-treated with curcumin and CGA to ensure full uptake and equilibration prior to LPS stimulation, helping to rule out effects, due to kinetic differences in their absorption versus LPS action. The cell culture supernatants were collected and stored at −80 °C until used for TNF-α quantification by ELISA. The remaining cell monolayer was immediately used to assess cytotoxicity via MTT assay or extracted for subsequent qRT-PCR analysis.

### 2.3. Quantification of TNF-α

The amount of TNF-α produced by the THP-1 cells in response to LPS was quantified using a Human TNF-α ELISA MAX^TM^ Deluxe Set Kit (BioLegend, San Diego, CA, USA) according to the manufacturer’s instructions.

### 2.4. Cell Viability

THP-1 cells treated with curcumin and CGA were washed with phenol red-free RPMI medium followed by incubation with 2 mL of MTT reagent (0.5 mg/mL) for 2 h at 37 °C. The washing was done thrice to remove any bioactive compound from the medium, thereby reducing their interference in MTT assay. After incubation, the MTT dye was removed, and the violet formazan crystals generated by the reduction of MTT were solubilised by adding 1 mL of acidified isopropanol for 20 min. The absorbance of the solution was then measured at 595 nm using a micro-plate reader (FLUOstar^®^ Optima, BMG Labtech^TM^, Ortenberg, Germany).

### 2.5. Quantitative Real-Time PCR (qRT-PCR)

Total RNA was extracted from curcumin, and CGA-treated THP-1 cells using an ISOLATE II RNA Mini Kit (Cat. # BIO-52073, Bioline, Auckland, NZ) and the RNA yield was determined by measuring the absorbance at 260/280 nm. cDNA was synthesised from 0.5 µg total RNA using a Reverse Transcription Kit (Cat. # 4368813, ThermoFisher Scientific^TM^, NZ). PCR reactions were set up using 1-µL cDNA template, 5-µl 2x SYBR^®^ green PCR master mix (Cat. # A25741, ThermoFisher, NZ) and 1 µL of gene-specific forward and reverse primers (10 µM each) in a final volume of 10 µL. PCR was performed for 40 cycles, with a denaturing step of 95 °C for 5 s followed by combined annealing and extension at 60 °C for 30 s using a StepOne Real-Time PCR instrument (ThermoFisher Scientific^TM^, Singapore). Primer sequences are listed in Table 1. Normalisation was performed using the housekeeping gene β-actin, and relative mRNA levels were expressed as fold changes relative to the control and calculated as ΔΔ*Ct* = 2^−(Δ*Ct_β-Actin_* − Δ*Ct_target gene_),* where, Ct is the cycle number at which the fluorescence signal of the reaction crossed the threshold.

### 2.6. Data Analysis

Statistical analysis was conducted using SPSS (IBM SPSS version 25). All data are presented as means± SD (standard deviation) unless otherwise specified, and the significance level for all statistical tests was set at *p* ≤ 0.05. The data were tested for normality using the Kolmogorov-Smirnov test. The results were expressed as mean ± standard deviation if the normality assumption was met. One-way analysis of variance (ANOVA) was used to determine significant differences between the means. Duncan’s post hoc comparisons were used to test for complementarity and/or synergy between curcumin and CGA. 

## 3. Results

### 3.1. Effect of Curcumin and CGA on TNF-α and Cell Viability

**Figure 1:**
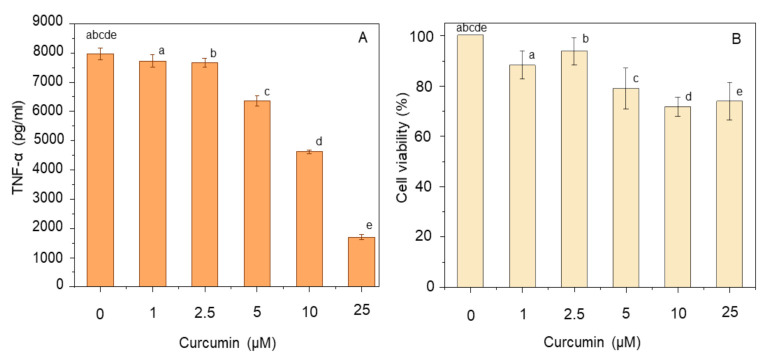
Effect of curcumin dose on TNF-α production (**A**) and cell viability (**B**). THP-1 macrophages were pre-treated with 1–25 µM curcumin for 1 h prior to stimulation with 100 ng/mL LPS for 4 h at 37 °C. The level of secreted TNF-α was measured using ELISA, and cell viability was assayed using MTT. Data represent the mean ± standard deviation for three biological replicates. One way ANOVA with Duncan’s post hoc test was used to perform the comparison of means. Means with the same letters are significantly different (*p* ≤ 0.05).

The anti-inflammatory potential of curcumin was tested in LPS-treated THP-1 macrophages by measuring the amount of TNF-α produced in an ELISA assay after incubation with different concentrations of curcumin (1–25 µM). Simultaneously, the effect of curcumin on cell viability was assessed by the MTT assay. In control cells (untreated with curcumin), but exposed to LPS, TNF-α was present in the medium at 7.8 ± 0.2 ng/mL (Figure 1A). Curcumin was effective in reducing this amount in a dose-dependent manner, with the level of TNF-α decreasing to 1.7 ± 0.1 ng/mL at 25 µM curcumin (Figure 1A). MTT assays revealed a small, dose-dependent loss of cell viability, decreasing to 70–75% at the two highest doses of curcumin (Figure 1B).

**Figure 2:**
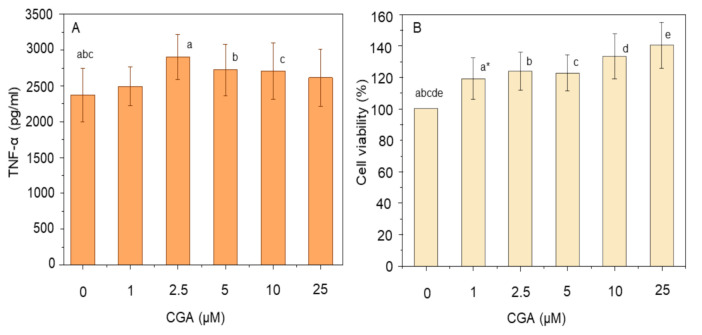
Effect of chlorogenic acid (CGA) dose on TNF-α production (**A**) and cell viability (**B**). THP-1 macrophages were pre-treated with 1–25 µM CGA for 1 h prior to stimulation with 100 ng/mL LPS for 4 h at 37 °C. The level of secreted TNF-α was measured using ELISA, and cell viability was assayed using MTT. Data represent the mean ± standard deviation for five * or six biological replicates. One way ANOVA with Duncan’s post hoc test was used to perform the comparison of means. Means with the same letter are significantly different (*p* ≤ 0.05).

In contrast to curcumin, treatment of THP-1 macrophages with CGA did not result in suppression of TNF-α release in response to LPS, but instead caused a small (yet significant) increase in TNF-α production (Figure 2A). As the concentration of CGA increased from 1 µM to 2.5 µM, the amount of TNF-α increased from 2.5 ± 0.3 ng/mL to 2.9 ± 0.3 ng/mL which was higher than the LPS-treated control (*p* ≤ 0.05). Simultaneously, a dose-dependent increase in cell viability was observed on pre-treating LPS-incubated THP-1 macrophages with CGA (Figure 2B). Over the range of concentrations used, there was a stepwise increase in cell viability up to 140.2 ± 14.6% at 25 µM, compared to the control (*p* ≤ 0.05). The increase in cell viability was even significant at a low dose of 1 µM (*p* ≤ 0.05).

**Figure 3:**
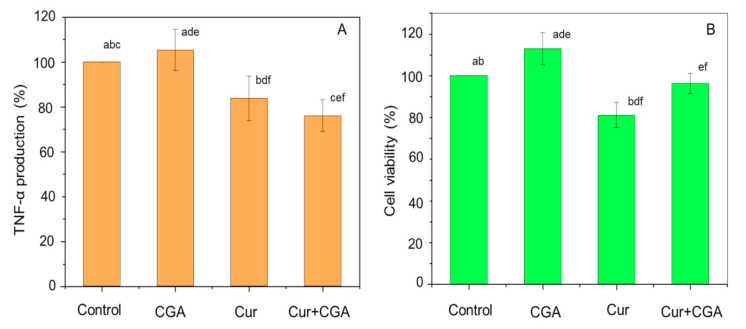
Effect of curcumin, chlorogenic acid (CGA) and their combinations on TNF-α production (**A**) and cell viability (**B**). THP-1 macrophages were pre-treated with 5 µM curcumin and CGA (alone and in combination) for 1 h prior to stimulation with 100 ng/mL LPS for 4 h at 37 °C. The level of secreted TNF-α was measured using ELISA, and cell viability was assayed using MTT. Data represent the mean ± standard deviation for nine biological replicates. One-way ANOVA with Duncan’s post hoc test was used to perform the comparison of means. Means with the same letter are significantly different (*p* ≤ 0.05).

To study the effect of combinations of curcumin with CGA, THP-1 cells were pre-treated with curcumin at a fixed concentration of 5 µM with varying concentrations of CGA (2.5–10 µM) for 1 h (results not shown). Cells were then challenged with LPS, and TNF-α and cell viability were measured. LPS-stimulated THP-1 cells (without bioactive compound), or treated with curcumin or CGA alone (prior to LPS-stimulation) were used as controls. Incubation with 5 µM curcumin caused a significant (*p* ≤ 0.05) reduction of ~16% in TNF-α release. Co-administration of curcumin with CGA also caused similar reductions in TNF-α secretion, but the decrease was generally less than that caused by 5 µM curcumin alone, except for cells treated with 5 µM curcumin and 5 µM CGA, where the level of TNF-α decreased by ~25%, from 4.0 ± 1.8 ng/mL to 3.0 ± 1.2 ng/mL (*p* ≤ 0.05) compared to curcumin alone (Figure 3A). In addition, for combination treatment, the cell viability was significantly higher than that of the 5 µM curcumin-treated control (*p* ≤ 0.05) (Figure 3B). Together, these data show that a combination of curcumin and CGA not only reduced TNF-α production, but also protects against cell death.

### 3.2. Effect of Curcumin and CGA on the NF-κB Signalling Pathway 

**Figure 4:**
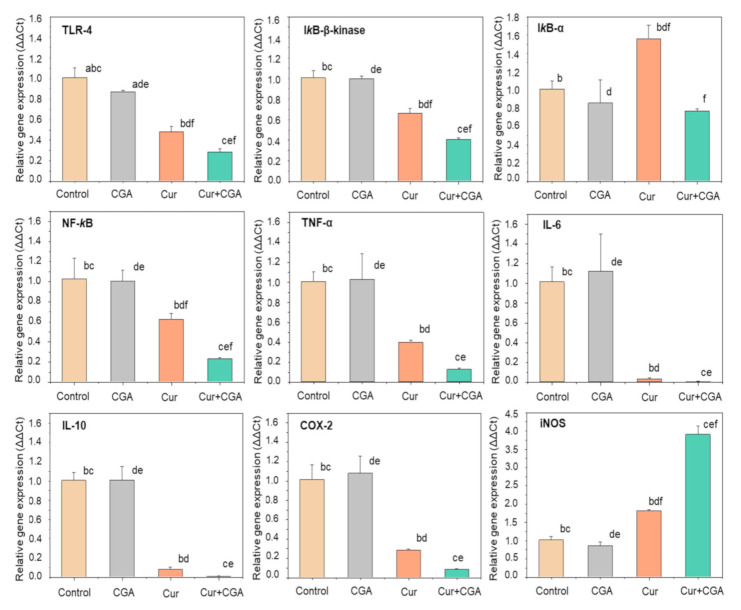
Effect of curcumin (Cur), chlorogenic acid (CGA) and their combination on mRNA expression of inflammatory biomarkers and NF-κB signalling pathway. THP-1 macrophages were pre-treated with curcumin, CGA and their 1:1 combination for 1 h prior to stimulation with 100 ng/mL LPS for 4 h at 37 °C. Expression of mRNAs was quantified using qRT-PCR. Cells treated with LPS, but not with curcumin and/or CGA were used as control. Data represent the mean ± standard deviation for three biological replicates. One-way ANOVA with Duncan’s post hoc test was used to perform the comparison of means. Means with the same letter are significantly different (*p* ≤ 0.05). ^a^ control-CGA; ^b^ control-Cur; ^c^ control-Cur+CGA, ^d^ CGA-Cur; ^e^ CGA-Cur+CGA; ^f^ Cur-Cur+CGA. TLR-4, toll-like receptor-4; IκB-α, inhibitor of κB-α; NF-κB, nuclear factor kappa-B; TNF-α, tumour necrosis factor-α; IL, interleukin; COX-2, cyclooxygenase-2; iNOS, inducible nitric oxide synthase.

To investigate the molecular mechanisms by which the combination of curcumin and CGA might exert their anti-inflammatory effects, we examined the mRNA expression of a variety of genes associated with inflammation, in particular, those involved in the NF-κB pathway and genes downstream of NF-κB. The relative mRNA levels of toll-like receptor-4 (TLR-4), IκB-α, IκB-β-kinase, NF-κB, TNF-α, IL-6, IL-10, COX-2 and iNOS were measured by qRT-PCR in THP-1 cells treated with either curcumin, CGA or both (Figure 4).

Quantitative RT-PCR, analysis revealed that except for a small suppression of TLR-4, CGA alone was not effective in reducing the mRNA expression of any of the inflammatory marker genes tested. In contrast, curcumin suppressed the expression of all of the genes tested, with the exception of iNOS. Even though treatment with CGA alone appeared to have little or no effect on THP-1 gene expression, the effect of curcumin was markedly potentiated by co-incubation with CGA in all cases, except where curcumin already had a large suppressive effect.

TLR-4 receptor expression was decreased by ~13% and ~52% with CGA and curcumin, respectively, but their combination suppressed it by ~72%. Curcumin alone downregulated the expression of IκB-β-kinase and NF-κB by ~34% and ~39%, respectively, and in combination with CGA, significant additional decreases of ~26% and ~38%, were observed, respectively (*p* ≤ 0.05). However, the combination of curcumin and CGA was not effective in reducing IκB-α expression. 

Treatment of the THP-1 cells with the combination of bioactive compounds was also able to downregulate the expression of NF-κB regulated genes. Expression of the pro-inflammatory cytokines, TNF-α and IL-6, was strongly suppressed by the combination treatment (~88% and ~99%, respectively), although this was not significantly different from the already strong suppression observed with curcumin alone. Surprisingly, curcumin and CGA had a similar effect in downregulating the expression of the anti-inflammatory cytokine, IL-10, although this might have been due to suppression of NF-κB, which plays a role in IL-10 expression.

Similarly, for the combination treatment, there was an additional reduction of ~20% in the mRNA level of COX-2, a downstream target of NF-κB and a key enzyme involved in arachidonic acid metabolism. In contrast, the expression of iNOS, an enzyme that initiates the production of bactericidal RNS in phagocytes, was upregulated by curcumin alone (~80%), with a very strong synergistic effect observed upon addition of CGA (~285%), even though CGA alone did not elicit an effect.

## 4. Discussion

The anti-inflammatory property of curcumin has been well-established and recognised as an asset to reduce chronic disease risk. The potential to enhance its anti-inflammatory property has been demonstrated by coupling with other bioactives, such as piperine, or using lipid-based delivery systems. The results presented in this study demonstrate the efficacy of CGA to potentiate the inflammation-lowering effects of curcumin in THP-1 cells. Our findings provide evidence of a complementary and synergistic reduction in mRNA levels of NF-κB, COX-2 and an increase in the expression of iNOS mRNA following concomitant treatment of cells with curcumin and CGA.

Bioactive compounds are naturally obtained from food sources and can be consumed as part of a normal diet rather than being consumed as a drug. However, in food, these compounds are available in small quantities which may not be sufficient to elicit the desired anti-inflammatory effects [33]. Therefore, combinations of different bioactives may be useful to achieve synergistic effects on inflammation. In this study, administration of curcumin prior to inducing an inflammatory response in macrophages caused a decrease in the production of TNF-α, a pro-inflammatory cytokine, in a dose-dependent manner, although with slight toxicity at the highest doses (Figure 1). This is consistent with previous observations where curcumin was effective in reducing TNF-α production with a decrease in cell viability [34,35]. Curcumin exhibits anti-inflammatory properties by influencing the activity of certain enzymes (e.g., COX-2 and iNOS), growth factors (e.g., transforming growth factor-β1—TGF-β1), receptors (e.g., integrin receptor—IR and interleukin 8-receptor—IL-8-R), kinases (e.g., mitogen-activated protein kinase—MAPK; and Janus kinase—JAK), inflammatory cytokines (e.g., TNF-α, IL-6 and IL-8) and transcription factors (e.g., NF-κB and nuclear factor erythroid 2-related factor—Nrf-2) [15,16].

In contrast, CGA is an antioxidant which shows anti-inflammatory effects predominantly by scavenging ROS and RNS, thereby reducing oxidative stress in the cell [29,30]. This decrease in oxidative stress will reduce the activation of the NF-κB signalling pathway and thereby reduce the production of various pro-inflammatory cytokines, such as TNF-α, and other cell mediators; ultimately reducing chronic inflammation in the host [36]. However, in our study, a small increase in the production of TNF-α was observed after treating THP-1 macrophages with CGA (Figure 2A). This increase in TNF-α production is most likely explained by the observed increase in cell viability (Figure 2B). Similar protective effects of CGA on cell viability have been reported previously [37,38,39,40]. It is possible that CGA can modulate apoptotic genes in LPS stimulated THP-1 macrophages as CGA has been shown to increase the production of B-cell lymphoma-2 (Bcl-2) and decrease the production of Bax, anti-apoptotic and pro-apoptotic proteins, respectively, and decrease caspase-3 in β-amyloid stimulated PC12 cells within 1 h of treatment [41]. To the best of our knowledge, our study is the first to demonstrate the protective effect of CGA in human immune cells. Further research is needed to identify the possible mechanisms responsible for THP-1 cell protection. We also found that co-administration of CGA with curcumin, augmented the effect of curcumin in reducing TNF-α whilst maintaining greater than 95% cell viability. In summary, pre-treatment of THP-1 cells with CGA alone improved cell viability without any reduction in TNF-α production, indicating its low anti-inflammatory activity. While curcumin treatment alone slightly reduced cell viability, its co-administration with CGA improved cell survival along with enhanced suppression of TNF-α production (Figure 3), showing that the combination of curcumin with CGA is better than either alone. Previous reports have also shown a similar augmented effect when combining curcumin (10 µM) with resveratrol (10 µM) in human articular chondrocytes [42].

**Figure 5:**
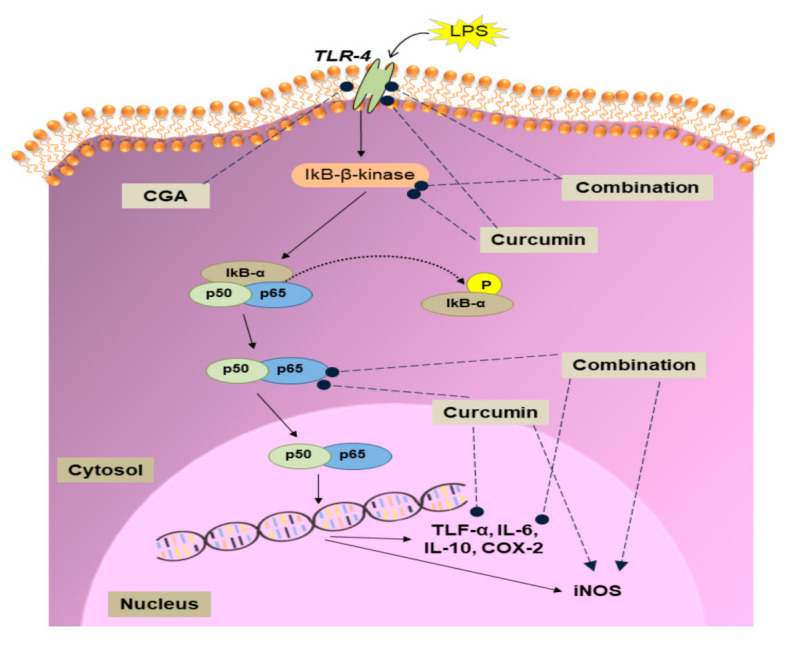
Summary of the effects of curcumin, chlorogenic acid (CGA) and their combination on the NF-κB signalling pathway. LPS, lipopolysaccharide; TLR-4, toll-like receptor-4; IκB-α, inhibitor of κB-α; TNF-α, tumour necrosis factor-α; IL, interleukin; COX-2, cyclooxygenase-2; iNOS, inducible nitric oxide synthase. Upregulation (►) and downregulation (●) of mRNA levels.

Along with TNF-α, several other pro-inflammatory cytokines (e.g., IL-6), anti-inflammatory cytokines (e.g., IL-10), and enzymes (e.g., COX-2 and iNOS) are also upregulated in response to inflammatory stimuli. The expression of these genes can be controlled by a variety of signalling pathways; including the JNK pathway, p38 MAPK pathway, P13k/Akt pathway and the NF-κB pathway [43]. NF-κB is the principal regulator influencing the expression of more than 500 inflammation-related genes [44]. We, therefore tested the effect of curcumin, CGA and their combination on the mRNA expression of various genes in the NF-κB signalling pathway. In unstimulated cells, the NF-κB protein is present in the cytoplasm and translocates into the nucleus upon activation [45]. In its inactive form, the NF-κB heterodimer is sequestered in the cytoplasm by binding with IκB, an NF-κB inhibitory protein [46]. On exposing the cells to LPS, the TLR-4 receptor is activated causing activation of IκB kinase (IKK), which phosphorylates IκB, targeting it for proteolytic degradation, thereby releasing NF-κB [47], which translocates into the nucleus to increase the transcription of various inflammation-related genes including TNF-α, IL-6, COX-2 and iNOS [46,48]. Our results show that treatment of macrophages with CGA and curcumin in combination resulted in an enhanced anti-inflammatory potential compared to curcumin alone, possibly by suppressing genes in the NF-κB pathway at the mRNA level, likely leading to decreased activity of the pathway. The combination was effective in synergistically downregulating the mRNA levels of both the TLR-4 receptor, and IκB kinase. The combination of curcumin with CGA also synergistically suppressed NF-κB expression. However, the reduction in expression of genes regulated by NF-κB (TNF-α, IL-6 and IL-10) by both compounds together was not significantly different from that seen with curcumin treatment alone. This was very probably due to the fact that these genes were almost fully suppressed by curcumin, leaving little room for an additional effect of CGA. It was expected that the combination treatment would enhance the expression of IL-10, an anti-inflammatory cytokine, but the opposite was observed. This reduction in IL-10 mRNA could be due to suppression of its regulator NF-κB. A similar reduction in COX-2 mRNA was observed on treatment with the combination. The effect of curcumin, CGA and their combination on the mRNA levels of NF-κB signalling pathway components is summarised in Figure 5. Further work will be required to determine if other inflammatory signalling pathways, such as p38-MAPK and JNK, are affected in a similar way.

## 5. Conclusions

Using an in vitro model of inflammation, in which THP-1 macrophages were stimulated with LPS, we have shown that curcumin and CGA synergistically reduce the production of the pro-inflammatory cytokine TNF-α. This combination of compounds was also effective in synergistically down-regulating the genes for pro-inflammatory cytokines and the COX-2 enzyme. These effects are likely explained by suppression of NF-κB pathway activity, due to decreased mRNA levels of NF-κB pathway components. Our pre-clinical study, conducted in cultured human cells, provides strong evidence to suggest that combining curcumin and CGA may be an effective new strategy to combat chronic inflammation. However, experimental findings in vitro do not necessarily translate directly to the situation in vivo. Further studies, including trials in animals and humans, should be conducted to confirm the effectiveness of this combination of compounds in the therapeutic suppression of inflammation.

## Figures and Tables

**Table 1:** Primer sequences used for qRT-PCR.

Gene	Accession Number	Primer Sequences	Product Size (BP)
IL-6	NM_001318095.1	F: TGGCAGAAAACAACCTGAACC	89
		R: TTTCACCAGGCAAGTCTCCTCAT	
IL-10	NM_000572.2	F: GTGATGCCCCAAGCTGAGA	138
		R: CACGGCCTTGCTCTTGTTTT	
TNF-α	NM_000594.2	F: CTGCTGCACTTTGGAGTGAT	93
		R: AGATGATCTGACTGCCTGGG	
iNOS	NM_000625.3	F: CATCCTCTTTGCGACAGAGAC	118
		R: GCAGCTCAGCCTGTACTTATC	
COX-2	NM_000963.3	F: TCCCTTGGGTGTCAAAGGTAAAA	144
		R: AACTGATGCGTGAAGTGCTG	
TLR-4	NM_003266.3	F: GGTCAGACGGTGATAGCGAG	180
		R: TTTAGGGCCAAGTCTCCACG	
IκB-α	NM_020529.2	F: AAGTGATCCGCCAGGTGAAG	281
		R: CGTGTGGCCATTGTAGTTGG	
IκB-β kinase	NM_001556.3	F: TCCGATGGCACAATCAGGAAA	264
		R: GCAGACCACAGCAGTTCTCA	
NF-κB	NM_003998.2	F: TGAGTCCTGCTCCTTCCA	103
		R: GCTTCGGTGTAGCCCATT	
β-Actin	NM_001101.4	F: CACTCTTCCAGCCTTCCTTC	104
		R: GTACAGGTCTTTGCGGATGT	
